# Lipid-Enriched Gintonin from Korean Red Ginseng Marc Alleviates Obesity via Oral and Central Administration in Diet-Induced Obese Mice

**DOI:** 10.3390/nu17233794

**Published:** 2025-12-03

**Authors:** Tamanna Yasmin, Yuna Lee, Won Seok Kim, Bonggi Lee, Rami Lee, Hongik Hwang, Min-Ho Nam, Seung-Yeol Nah, Min Soo Kim, Hyewhon Rhim

**Affiliations:** 1 Brain Science Institute, Korea Institute of Science and Technology, Seoul 02792, Republic of Korea; 622002@kist.re.kr (T.Y.);; 2 Division of Bio-Medical Science & Technology, KIST School, University of Science and Technology (UST), Seoul 02792, Republic of Korea; 3 Department of Life Science, Korea University, Seoul 02841, Republic of Korea; 4 Department of Food Science and Nutrition, Pukyong National University, Busan 48513, Republic of Korea; bong3257@pknu.ac.kr; 5 Ginsentology Research Laboratory and Department of Physiology, College of Veterinary Medicine, Konkuk University, Seoul 05029, Republic of Korea; 6 Department of Life Science, University of Seoul, Seoul 02504, Republic of Korea; 7 KHU-KIST Department of Converging Science and Technology, Kyung Hee University, Seoul 02447, Republic of Korea

**Keywords:** Korean red ginseng marc, gintonin, obesity, brown adipose tissue, metabolism, thermogenesis

## Abstract

**Background:** Korean red ginseng marc (KRGM), a by-product of Korean red ginseng (KRG) processing, retains numerous bioactive compounds with potential health benefits. Among them, KRGM-derived gintonin (KRGM-gintonin) is particularly rich in lysophosphatidic acid (LPA) and phospholipids, which have been linked to favorable metabolic effects. This study investigated the anti-obesity potential of KRGM-gintonin in high-fat diet (HFD)–induced obese mice, focusing on its impact on weight regulation, liver health, and energy metabolism. **Methods:** Obese mice (C57BL/6N, 4 weeks, male) were administered KRGM-gintonin either orally for 25 weeks or through intracerebroventricular (ICV) injection for 14 weeks. Throughout the study, body weight, food intake, metabolic parameters, liver tissue morphology, behavioral performance, and thermogenic gene expression were carefully monitored to evaluate treatment effects. **Results:** Both oral and ICV administration of KRGM-gintonin significantly reduced body weight gain in HFD-fed obese mice without altering food intake, suggesting enhanced energy expenditure. Treatment through both routes improved physical performance and increased metabolic rate. Oral KRGM-gintonin also alleviated fatty liver, reduced plasma triacylglycerol and cholesterol levels, and promoted the expression of thermogenesis-related genes, including uncoupling protein-1 (UCP1) and hormone-sensitive lipase (HSL), specifically in brown adipose tissue. Additionally, oral administration lowered tumor necrosis factor-α (TNF-α) expression, indicating anti-inflammatory activity and further supporting metabolic health. **Conclusions:** KRGM-gintonin exerts strong anti-obesity effects, primarily through oral administration, with supportive evidence from central ICV action. These findings highlight its potential as a functional therapeutic agent for obesity prevention and management, offering dual benefits in metabolic regulation and inflammation control.

## 1. Introduction

Obesity is a pervasive global health concern associated with an increased risk of severe comorbidities, including cardiovascular disease, type 2 diabetes mellitus, and certain cancers. It arises from a chronic imbalance between energy intake and expenditure, leading to the excessive accumulation of adipose tissue [1,2]. Consequently, developing effective strategies to enhance energy expenditure and reduce adiposity is essential for the management of obesity.

Adipose tissue plays a central role in maintaining metabolic homeostasis [3]. In mammals, white adipose tissue (WAT) primarily stores excess energy as triglycerides, whereas brown adipose tissue (BAT) dissipates energy through non-shivering thermogenesis [4]. BAT is rich in mitochondria and expresses uncoupling protein-1 (UCP1), which uncouples oxidative phosphorylation to generate heat rather than ATP [5,6]. Notably, WAT can undergo “browning,” acquiring BAT-like features including elevated mitochondrial content and UCP1 expression, thereby increasing energy expenditure [6,7]. Activation of thermogenic pathways, particularly via cAMP-mediated signaling that upregulates UCP1, PRD1-BF1-RIZ1 homologous domain containing 16 (PRDM16), and PGC1α, represents a promising therapeutic approach for obesity [5,8,9].

Growing interest has centered on bioactive natural compounds capable of enhancing thermogenesis and modulating adipose tissue metabolism. Korean red ginseng (KRG) is well recognized for its medicinal properties, including anti-obesity and metabolic benefits [10,11]. During the production of Korean Red Ginseng (KRG), a substantial residual solid byproduct known as Korean Red Ginseng marc (KRGM) is generated after the water extraction process; this marc represents the leftover fibrous material after water extraction, accounting for approximately 40–50% of the raw material, and contains valuable unextracted bioactive components including gintonin, lipids, and minor ginsenosides. While ginsenosides have been extensively studied, gintonin, a glycolipoprotein complex, has recently attracted attention for its receptor-mediated biological activities, including the regulation of calcium signaling and metabolic processes [12,13,14].

Traditionally, gintonin has been extracted from fresh ginseng root or whole extracts. The Korean ginseng (*Panax ginseng* C.A. Meyer, family *Araliaceae*) industry produces approximately 8000 tons of Korean red ginseng marc (KRGM) annually, much of which remains underutilized despite containing substantial amounts of bioactive components, including gintonin, lipids, polyphenols, proteins, fiber, and minor ginsenosides [15]. The isolation procedure of KRGM-derived gintonin ensures enrichment of these bioactive phospholipids and lysophospholipids (including LPA, LPC, and PC), while simultaneously valorizing this abundant byproduct as a sustainable and cost-effective source of functional compounds. Table 1 summarizes the differences in major lipid components between KRGM-gintonin and conventional gintonin-enriched fraction (GEF), highlighting the higher content of bioactive phospholipids and lysophospholipids in KRGM-gintonin. These compositional distinctions suggest that KRGM-gintonin may exert superior anti-obesity and thermogenic effects compared to conventional GEF [15,16]. Previous studies have demonstrated that KRG and its derivatives exert anti-obesity effects through multiple mechanisms, including inhibition of adipogenesis and lipogenesis, enhancement of lipolysis, and promotion of thermogenesis in adipose tissues [17,18]. The remaining ginsenosides and lipid-bound LPAs in red ginseng are considered to serve as key precursors for gintonin, a bioactive glycolipoprotein complex that can regulate energy metabolism and lipid homeostasis [14,16].

Recent studies have also highlighted KRGM-gintonin’s potential neuroprotective and skin anti-aging effects [15,19]. Moreover, gintonin has been shown to suppress adipocyte differentiation and improve glucose metabolism both in vitro and in vivo [20]. Specifically, GEF enhances lipolysis and energy expenditure by elevating intracellular calcium and activating LPA receptor signaling, which upregulates thermogenic genes such as UCP1, PRDM16, and PGC1α [20,21]. Despite these promising findings, the anti-obesity efficacy of KRGM-derived gintonin itself remains largely unexplored.

Therefore, the present study is the first to investigate the long-term effects of KRGM-gintonin administered via oral and intracerebroventricular (ICV) routes using an alternate-day dosing regimen over 25 and 14 weeks, respectively. Unlike previous studies that employed short-term daily administration (6 weeks) of GEF extracted from ginseng [21], our design enables the evaluation of both peripheral and central contributions to energy metabolism and body weight regulation. We hypothesize that the enriched bioactive lipid profile of KRGM-gintonin confers enhanced anti-obesity efficacy through upregulated activation of thermogenic signaling pathways, underscoring its potential as a sustainable and upcycled by-product.

## 2. Materials and Methods

### 2.1. Materials

The Korean Red Ginseng marc (KRGM) was provided and verified by the Ginsentology Research Laboratory, Konkuk University (Seoul, Republic of Korea). The KRGM-gintonin used in this study was prepared according to a previously established protocol, and its identity was confirmed by LC-MS/MS lipid profiling [15]. The lipid composition of KRGM-gintonin and conventional gintonin-enriched fraction (GEF) shown in Table 1 was adopted from these reports [16]. All reagents were obtained from Sigma-Aldrich (St. Louis, MO, USA), except ethanol, which was supplied by Korea Ethanol Supplies Company (Seoul, Republic of Korea).

### 2.2. Experimental Animals and Treatments

#### 2.2.1. Animals

A total of 64 male C57BL/6N mice, four weeks old and weighing 18–21 g, were obtained from DBL Co., Ltd. (Eumseong-gun, Chungcheongbuk-do, Republic of Korea). The animals were housed under controlled conditions: a temperature of 23 ± 1 °C, relative humidity of 50 ± 10%, and a 12 h light/dark cycle, with ad libitum access to food and water. Upon arrival, the mice underwent a one-week acclimatization period in the animal facility at the Korea Institute of Science and Technology (KIST). All procedures were approved by the Institutional Animal Care and Use Committee (IACUC) of KIST (approval number: KIST-2022-012). The animal cages were located in the same room, but the cage location did not rotate.

To reduce postoperative pain after stereotaxic surgery for ICV cannula implantation, meloxicam (Metacam, Boehringer Ingelheim, Seoul, Republic of Korea) was injected subcutaneously at 2 mg/kg body weight. Body weight loss, injury, vitality and unconsciousness were observed for humane endpoints. No mice met the humane endpoint criteria. Metabolic rate, behavioral tests, and body composition were assessed after at least 12 weeks of oral administration. Animals were anesthetized with isoflurane and subsequently euthanized by cervical dislocation. Blood samples and organ tissues were obtained upon sacrifice at the end of the experiment for subsequent biochemical and histological analyses.

Animals were randomly assigned to groups. Investigators administering oral gavage or ICV injections were necessarily aware of the allocation. Grip strength testing was performed by a blinded evaluator, whereas body weight, biochemical, and histological analyses were conducted unblinded by the research team.

#### 2.2.2. Oral Administration

All mice (44) were randomly assigned to four groups, each consisting of 10 animals, for oral administration of KRGM-gintonin. The groups were as follows: a chow diet (CD) group treated with PBS (Control) and three groups fed a high-fat diet (HFD; Research Diets D12492) containing 60% kcal (diet composition is shown in Supplementary Table S1) from fat. Among the groups, mice received either PBS, KRGM-gintonin at 50 mg/kg, or KRGM-gintonin at 200 mg/kg, administered with Zonde three times per week. Throughout the experimental period, body weight was measured weekly at 11:00 AM using a calibrated analytical balance. Food intake was measured over 24 h on four separate days for 3 months, and no significant differences were identified in cumulative or average food intake among the experimental groups

Previous studies using GEF have reported oral doses ranging from 50 to 150 mg/kg/day, depending on the experimental model [21]. Accordingly, the oral doses used in the present study (50 and 200 mg/kg) fall within or slightly above this range and were selected to represent moderate and high dosing regimens. To evaluate long-term effects, these doses were administered on alternate days for a total of 25 weeks.

#### 2.2.3. ICV Injection

Stereotactic surgery was performed to implant a guide cannula following standard protocols [22] allowing precise ICV administration of KRGM-gintonin to investigate its functional effects on obesity-related parameters. An animal stereotaxic apparatus (Kopf Instruments, Tujunga, CA, USA) was used to accurately position a 26-gauge guide cannula (Plastics One, Roanoke, VA, USA) at coordinates 2.0 mm posterior to the bregma and 5.0 mm below the bregma. Cannulation surgery was performed on all animals, regardless of treatment group.

For the ICV administration experiment, an additional cohort of 20 mice was used and randomly divided into three groups *(n* = 6–7 per group): Control (chow diet + PBS), HFD + PBS, and HFD + KRGM-gintonin (30 ng/0.5 µL). This study is the first to investigate the central effects of KRGM-derived gintonin through ICV administration into the third ventricle (3V) of the hypothalamus via a stereotaxically implanted cannula, with treatments administered three times per week for 14 weeks. The ICV administration approach was adapted from a previously reported protocol utilizing gintonin to target the central nervous system [22]. This regimen aimed to ensure sustained central exposure without inducing adverse neurological effects.

### 2.3. Analysis of Metabolic Rates and Energy Expenditure Profiles

To evaluate metabolic rates and related parameters, all mice were assessed using indirect calorimetry, following previously described methods [8], with the Comprehensive Laboratory Animal Monitoring System (CLAMS; Columbus Instruments, Columbus, OH, USA). Mice were individually placed in transparent respiratory chambers (20.5 × 10.5 × 12.5 cm). Over a 24 h period, measurements were collected for carbon dioxide (CO_2_) production, oxygen (O_2_) consumption, energy expenditure, heat production, and activity levels.

Data were acquired using Oxymax for Windows software (version 5.40.14; Columbus Instruments) and analyzed with CLAX software (version 2.2.15; Columbus Instruments). The respiratory exchange ratio (RER; CO_2_/O_2_) and delta-heat values were calculated from the recorded data using CLAX software.

### 2.4. Behavioral Tests

Animals were allowed to habituate for at least 1 h prior to the behavioral experiments, which were conducted after a minimum of 12 weeks of oral KRGM-gintonin treatment.

#### 2.4.1. Exhaustive Running Test

The duration of exhaustive running was measured using a treadmill (Jeung Do BIO & Plant Co., Ltd., Seoul, Republic of Korea). Prior to testing, mice underwent a 4-day acclimation period on the treadmill, running at a speed of 9 m/min on a 5° incline. On the fifth day, the exhaustive running test was conducted. The test began at an initial speed of 9 m/min on the same incline, with the speed increasing by 3 m/min every 3 min until reaching a maximum of 33 m/min. The trial was terminated when a mouse remained off the treadmill belt for more than 20 s and simultaneously exhibited a marked reduction in responsiveness to external stimuli. At this point, the mouse was gently removed from the treadmill, and the duration of exhaustive running was recorded.

#### 2.4.2. Open Field Test

Mice were habituated to handling for 10 min per day over three consecutive days. On the following day, each mouse was individually placed in an open-field arena (40 × 40 × 40 cm), and locomotor activity was monitored for 10 min under a light intensity of 50 lux. Behavior was recorded from a top view using a video camera and subsequently analyzed with Any-maze software version 7.48 (Stoelting Co., Wood Dale, IL, USA).

#### 2.4.3. Rotarod Test

The rotarod test was used to assess sensorimotor performance, balance, and motor coordination in mice. Assessments were conducted using a rotarod apparatus (BS Techno Lab, Inc., Seoul, Republic of Korea). At the start of each session, the rod was set to rotate at 2 rpm. Groups of four mice were placed simultaneously on the rod, each in an assigned position. Over a 5 min testing period, the rotation speed gradually accelerated to a final speed of 40 rpm. When a mouse fell from the rod, it interrupted an infrared beam, which automatically stopped both the rotation and the timer. The latency to fall (time spent on the rod without falling) was recorded, with a maximum cut-off time of 5 min. Each mouse underwent three trials per day over three consecutive days, with a 30 min rest interval between trials to minimize fatigue. To reduce the risk of injury from falls, a cushioned pad was placed beneath the apparatus.

#### 2.4.4. Forelimb Grip Strength Test

Forelimb grip strength was measured using a Grip Strength Meter (FGP-5, Jeung Do BIO & Plant Co., Ltd., Seoul, Republic of Korea). The digital force transducer recorded the maximum tensile force exerted by each mouse grasping the bar, expressed in grams. Three measurements were taken per day for each mouse, with one-minute intervals between trials. The order of testing was randomized daily, and the evaluator was blinded to previous results. Testing was performed in the vivarium during the light cycle between 11:00 AM and 5:00 PM. The device was regularly calibrated by the manufacturer to ensure accuracy.

### 2.5. Biochemical Assays

Biochemical analyses were performed after the completion of the in vivo experiments. The plasma triglyceride (TG) and total cholesterol (TC) levels were quantified using enzymatic assay kits (Asan Pharm., Seoul, Republic of Korea) according to the manufacturer’s instructions. Plasma free fatty acid (FFA) concentrations were measured using the acyl-CoA synthetase–acyl-CoA oxidase (ACS-ACOD) method with the NEFA-HR reagent kit (Wako, Tokyo, Japan), following the manufacturer’s protocol.

### 2.6. Body Composition Analysis

Body composition was assessed in mice at 24 weeks of age using an Echo-MRI-100 system (Echo Medical Systems, Waco, TX, USA) to determine fat and lean mass. Measurements were conducted at the Korea Model Animal Priority Center (Seoul, Republic of Korea).

### 2.7. Histology

Liver and tibialis anterior (TA) muscle tissues were fixed overnight in 10% formalin at 4 °C. Following fixation, tissues were cryoprotected by immersion in 30% sucrose solution before processing. For histological analysis, liver samples were frozen, sectioned, and air-dried on slides, while both liver and TA muscle tissues were embedded in paraffin for sectioning. Sections were stained with hematoxylin and eosin (H&E) and Oil Red O (ORO) to evaluate tissue morphology and lipid accumulation, respectively. Formalin fixation was used as the primary tissue preservation method, followed by rinsing of slides in water prior to staining. Histological procedures were performed at Genoss (Suwon-si, Gyeonggi-do, Republic of Korea).

### 2.8. Total RNA Isolation, cDNA Synthesis, and Quantitative Real-Time PCR

Total RNA was extracted from adipose tissue and liver following the manufacturer’s protocol using TRIzol reagent (Invitrogen Life Technologies, Waltham, MA, USA). For tissue homogenization, 1.0 mL of TRIzol solution was added per 100 mg of tissue. After thorough homogenization, 0.2 mL of chloroform was added to the mixture, which was then incubated at room temperature. The samples were centrifuged at 12,000× *g* for 15 min at 4 °C to separate the phases. The aqueous phase was carefully transferred, and total RNA was precipitated by adding 0.5 mL of isopropanol, followed by incubation at ambient temperature. Subsequently, the samples were centrifuged again at 12,000× *g* for 10 min at 4 °C. The resulting white, gel-like RNA pellet was washed with 75% ethanol and centrifuged at 7500× *g* for 5 min at 4 °C. The RNA pellet was then resuspended in diethylpyrocarbonate (DEPC)-treated water, and RNA concentration was quantified using a NanoDrop spectrophotometer (Thermo Scientific, Waltham, MA, USA). Complementary DNA (cDNA) synthesis was performed using a kit from Takara Bio USA, Inc. (San Jose, CA, USA), via reverse transcription of 5 µg total RNA. Quantitative real-time PCR (qRT-PCR) analysis was carried out using Power SYBR^®^ Green PCR Master Mix on a QuantStudio™ 3 Real-Time PCR System (Thermo Scientific). Primer sequences used in this study are listed in Table 2. Beta-actin was used as the internal reference gene to normalize target gene expression levels.

### 2.9. Statistical Analysis

Experimental data are expressed as mean ± standard error of the mean (SEM). In some treatments, the SEM values were very small, which may result in barely visible or invisible error bars in the figures. Statistical analyses were conducted using one-way analysis of variance (ANOVA) followed by Tukey’s post hoc test to assess differences among groups. Data analysis and graphical representation were carried out using GraphPad Prism 10 software (GraphPad Software Inc., Boston, MA, USA). Statistical significance was defined as * *p* < 0.05, ** *p* < 0.01, *** *p* < 0.001, **** *p* < 0.0001.

## 3. Results

### 3.1. Oral Administration of KRGM-Gintonin Exhibits Anti-Obesity Effects in HFD-Induced Obese Mice

**Figure 1:**
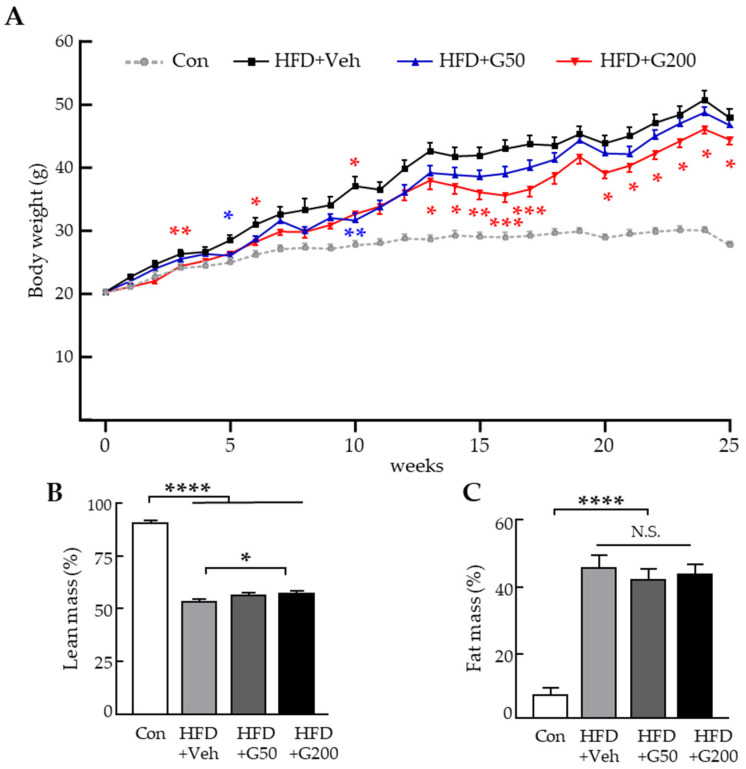
The administration of KRGM-gintonin reduces body weight and increases lean mass in high-fat diet (HFD)-fed mice. (**A**) Body weight was measured weekly during 25 weeks of KRGM-gintonin treatment. (**B**) Lean mass and (**C**) fat mass were assessed at week 24 using Echo-MRI. Data are expressed as mean ± S.E.M. (*n* = 10–11). Statistical significance between control (Con), HFD+Veh, HFD+G50 (KRGM-gintonin, 50 mg/kg), and HFD+G200 (KRGM-gintonin, 200 mg/kg) were determined using one-way ANOVA followed by Tukey’s post hoc test: * *p* < 0.05, ** *p* < 0.01, *** *p* < 0.001, **** *p* < 0.0001. N.S., not significant. Asterisks in different colors denote statistically significant differences between the respective treatment groups indicated by matching colors (e.g., red asterisks for group HFD+G200 blue asterisks for group HFD+G50).

To investigate the in vivo anti-obesity effects of KRGM-gintonin, 4-week-old male C57BL/6N mice were used. The animals were divided into groups and fed either a standard chow diet (Con) or a high-fat diet (HFD; 60% kcal from fat). Mice received oral administration of either PBS (Con and HFD groups), a low dose of KRGM-gintonin (HFD+G50; 50 mg/kg), or a high dose (HFD+G200; 200 mg/kg), three times per week for 25 weeks. Mice treated with KRGM-gintonin for 25 weeks exhibited protection against body mass gain (Figure 1A, tabular data in Supplementary Table S2). Specifically, body weight in the KRGM-gintonin–treated groups was significantly lower than in the HFD group. Mice fed HFD alone gained 47.9 ± 3.8 g over 25 weeks, whereas those treated with KRGM-gintonin gained 46.7 ± 2.4 g with HFD+G50 and 44.4 ± 2.5 g with HFD+G200. Importantly, the reduction in body weight induced by KRGM-gintonin was not attributable to decreased food intake, as no significant statistical differences were observed in food consumption among the diet groups (Figure S1). Body composition analysis using magnetic resonance imaging (MRI) further revealed a significant increase in lean-mass percentage in the HFD+G200 group compared to the HFD group, although fat mass did not change significantly (Figure 1B,C, tabular data in Supplementary Table S3). Collectively, these results suggest that long-term oral administration of KRGM-gintonin, particularly at the higher dose, effectively mitigates diet-induced weight gain and enhances lean body mass without affecting caloric intake, indicating its potential as a functional candidate for metabolic regulation.

### 3.2. Behavioral Assessments Reveal Improved Muscle Function Following Oral Administration of KRGM-Gintonin

**Figure 2:**
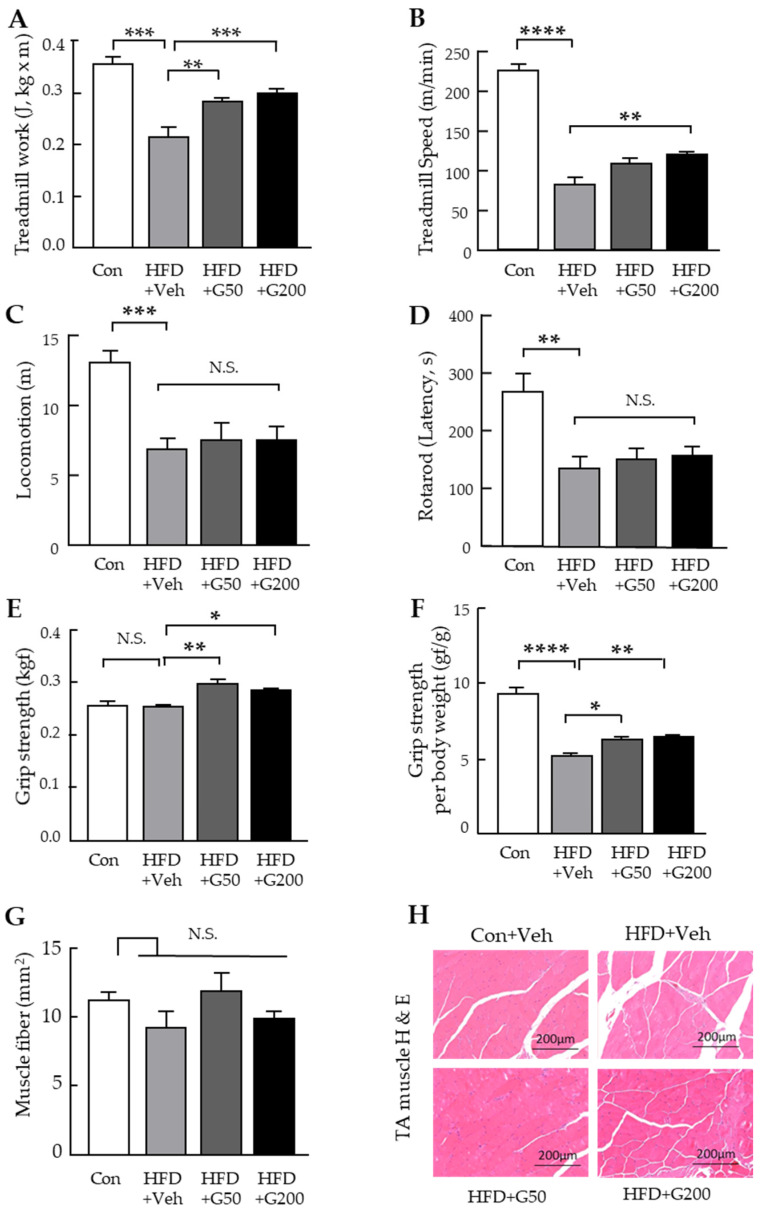
Effects of KRGM-gintonin on exercise performance and muscle function in HFD-fed mice. Mice were subjected to a treadmill running test with a 5° incline and increasing speed until exhaustion. (**A**) Total treadmill work performed and (**B**) maximum speed achieved. (**C**) Locomotor activity assessed by the open field test showed no significant change following KRGM-gintonin treatment. (**D**) Motor coordination and learning evaluated by the accelerating rotarod test were also unaffected by treatment. (**E**) Effect of KRGM-gintonin on normalized forelimb grip strength and (**F**) grip strength adjusted for body weight. (**G**) Quantification of tibialis anterior (TA) muscle analyzed by Image J v1.54r software. (**H**) Representative H&E staining of skeletal muscle (Scale bar: 200 μm, magnification: 10×). Data are expressed as mean ± S.E.M. (*n* = 4). Statistical differences between Con, HFD+Veh, HFD+G50, and HFD+G200 were determined using one-way ANOVA followed by Tukey’s post hoc test: * *p* < 0.05, ** *p* < 0.01 *** *p* < 0.001, **** *p* < 0.0001, N.S.: not significant.

We next investigated the effects of KRGM-gintonin on exercise performance in HFD-fed mice. The experimental subjects were 20-week-old mice. Prior to the exercise tolerance test, all mice were acclimated to treadmill running at a 5° incline for five consecutive days. Following acclimation, they were subjected to a treadmill running protocol at the same incline until exhaustion, defined as the inability to continue running for at least one minute despite mechanical stimulation. Both low and high doses of KRGM-gintonin significantly improved exercise performance compared with the HFD group (Figure 2A,B). Notably, mice treated with the high dose (HFD+G200) exhibited significantly enhanced endurance relative to both the untreated HFD group and the low-dose group (HFD+G50).

To evaluate spontaneous locomotor activity, an open field test was conducted by measuring the cumulative distance traveled over a 10 min period. KRGM-gintonin treatment did not produce significant changes in locomotor activity (Figure 2C). To further assess motor coordination and skill acquisition, 22-week-old mice were subjected to the accelerated rotarod test. No significant differences in motor performance were observed between KRGM-gintonin–treated and untreated groups (Figure 2D). At 23 weeks of age, grip strength testing revealed that KRGM-gintonin administration positively influenced muscle strength (Figure 2E). Specifically, grip strength normalized to body weight increased in a dose-dependent manner compared with the HFD group (Figure 2F). Although KRGM-gintonin significantly enhanced functional muscle performance, it did not substantially affect tibialis anterior (TA) muscle fiber size (Figure 2G). Histological analysis of TA muscle cross-sections showed no significant differences in muscle fiber size between the HFD and CD groups, or between the KRGM-gintonin–treated and HFD groups (Figure 2H). Collectively, these findings indicate that KRGM-gintonin enhances skeletal muscle function and exercise capacity without exerting major effects on general locomotor activity or muscle hypertrophy.

### 3.3. Oral Administration of KRGM-Gintonin Enhances Energy Expenditure and Alleviates Hepatic Steatosis in HFD-Fed Mice

**Figure 3:**
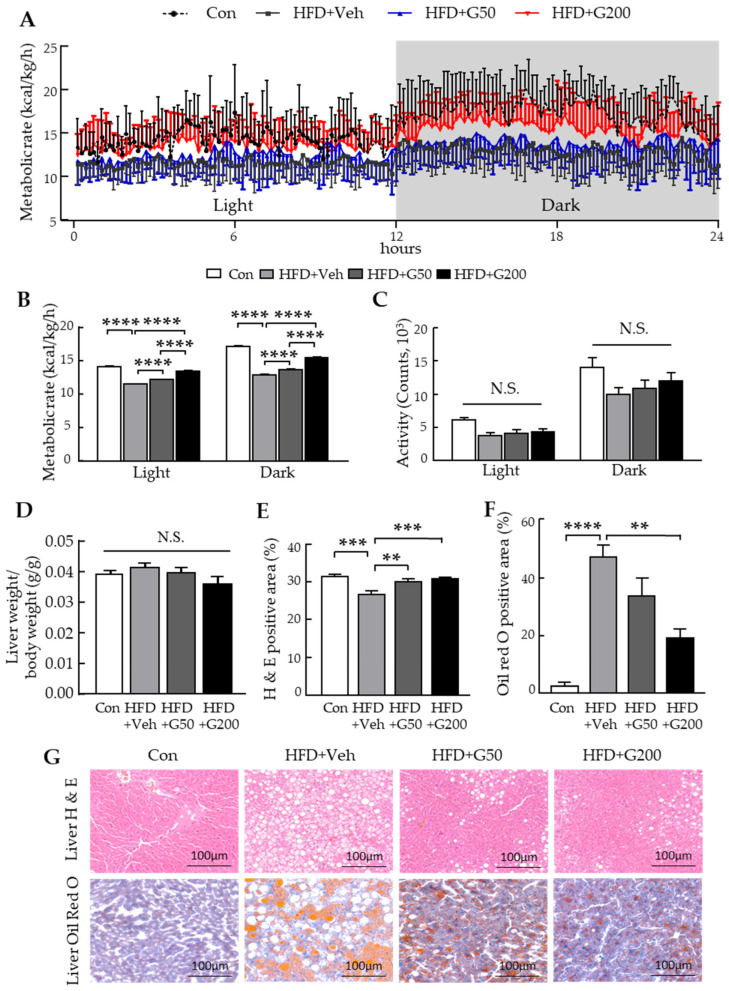
Oral administration of KRGM-gintonin regulates metabolic function in HFD-fed mice. (**A**) Energy expenditure was measured by indirect calorimetry in 18–20-week-old mice (*n* = 10–11) fed a chow diet, HFD with or without KRGM-gintonin oral treatment during the 12:12 h light and dark cycles. (**B**) The 24 h metabolic rate (kcal/kg/h) was calculated based on the oxygen consumption and carbon dioxide production. SEM values were very small, resulting in barely visible or invisible error bars. (**C**) Activity was counted for 24 h. (**D**–**G**) Oral administration of KRGM-gintonin attenuated hepatic lipid accumulation in HFD-fed mice (*n* = 4). (**D**) Liver weight was measured and expressed relative to body weight (%). (**E**) Hematoxylin and eosin (**H**, **E**) staining showing increased intracellular lipid deposition in the liver of HFD-fed mice. (**F**) Oil Red O (ORO) staining of liver sections to validate the lipid-lowering effect of KRGM-gintonin. (**G**) Representative images of H&E and ORO staining in liver sections at week 25 (scale bars: 100 μm; magnification: 10×). Statistical differences between Con, HFD+Veh, HFD+G50, and HFD+G200 were determined using one-way ANOVA followed by Tukey’s post hoc test: ** *p* < 0.01, *** *p* < 0.001, **** *p* < 0.0001, N.S., not significant.

Since KRGM-gintonin was shown to reduce body weight without affecting food intake, we hypothesized that it might enhance energy expenditure. To test this, 18–20-week-old mice (*n =* 10–11) were fed either a CD or HFD, with or without KRGM-gintonin treatment. Indirect calorimetry measurements were conducted across both light and dark cycles. Mice treated with low (HFD+G50) and high (HFD+G200) doses of KRGM-gintonin exhibited significantly increased metabolic rates during both light and dark phases (Figure 3A,B; Supplementary Table S7). This elevation in energy expenditure was not attributable to changes in physical activity (Figure 3C).

Histological analysis of liver tissue revealed no significant differences in relative liver weight among the CD, HFD, and KRGM-gintonin treated groups, although a non-significant decreasing trend was observed in the lower-dose treatment group (Figure 3D). H&E staining of liver sections showed a marked reduction in hepatic fat accumulation in both low- and high-dose KRGM-gintonin groups compared with the untreated HFD group (Figure 3E), indicating a protective effect against hepatic injury and steatosis.

To further evaluate hepatic lipid deposition, Oil Red O (ORO) staining was performed. Mice fed an HFD exhibited substantial intracellular lipid accumulation, whereas KRGM-gintonin–treated mice, particularly those in the HFD+G200 group, displayed a pronounced reduction in both the size and number of hepatic lipid droplets (Figure 3F). Additional microscopic examination of H&E- and ORO-stained liver sections confirmed a KRGM-gintonin–mediated attenuation of lipid droplet accumulation in hepatocytes (Figure 3G). Taken together, these findings suggest that KRGM-gintonin reduces HFD-induced body weight gain by enhancing energy expenditure and alleviates hepatic steatosis through the suppression of lipogenesis and hepatic lipid accumulation.

### 3.4. Alteration of Gene Related to Metabolic Parameters and Thermogenesis Signaling in BAT

**Figure 4:**
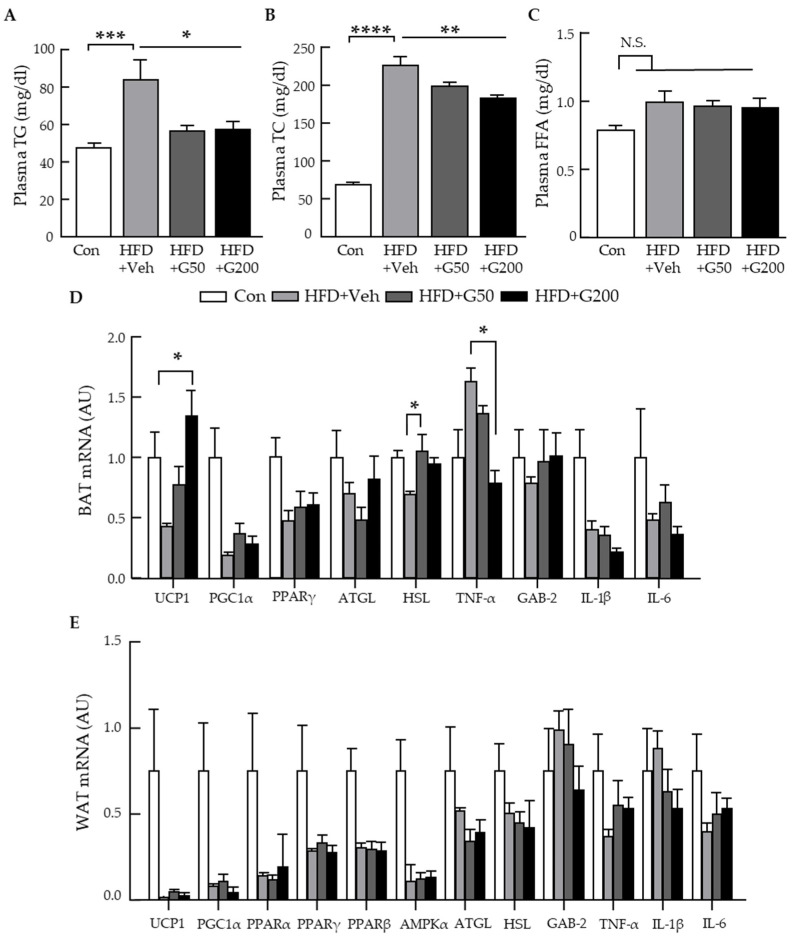
Oral administration of KRGM-gintonin improves the serum lipid profile and modulates thermogenic, lipolytic and inflammatory gene expression in brown adipose tissue (BAT) of HFD-fed mice. (**A**) Serum total triglycerides (TG), (**B**) total cholesterol (TC), and (**C**) free fatty acid (FFA) levels measured at the end of the study. (**D**) BAT and (**E**) white adipose tissue (WAT) samples (*n* = 4–6 per group) were collected for quantitative PCR analysis of thermogenesis-related gene expression. Statistical differences between Con, HFD+Veh, HFD+G50, and HFD+G200 were determined using one-way ANOVA followed by Tukey’s post hoc test: * *p* < 0.05, ** *p* < 0.01, *** *p* < 0.001, **** *p* < 0.0001, N.S., not significant.

To explore the relationship between long-term oral administration of KRGM-gintonin and improvements in metabolic features, we examined plasma lipid profiles. Treatment with low (HFD+G50) or high (HFD+G200) doses of KRGM-gintonin significantly reduced circulating levels of TG and TC, with no observable effect on free fatty acids (FFAs) (Figure 4A–C; tabular data in Supplementary Table S4). Next, we assessed whether KRGM-gintonin modulates the expression of genes associated with thermogenesis, lipolysis, and inflammation in adipose tissues using quantitative PCR. In BAT, the mRNA expression levels of thermogenic (e.g., UCP1) and lipolytic (e.g., HSL) genes were significantly upregulated in KRGM-gintonin–treated groups compared with the HFD group, whereas no such changes were observed in WAT (Figure 4D,E; tabular data in Supplementary Tables S5 and S6). These results indicate that high-dose KRGM-gintonin specifically promotes thermogenesis in BAT. To further investigate the anti-inflammatory potential of KRGM-gintonin, we quantified mRNA expression levels of inflammatory markers in BAT of HFD-fed mice. The expression of TNF-α was significantly reduced in the HFD+G200 group compared with the untreated HFD group, indicating that KRGM-gintonin suppresses inflammatory signaling in BAT. Overall, these findings support the conclusion that long-term oral supplementation with KRGM-gintonin enhances thermogenic and lipolytic gene expression while reducing inflammation in BAT in a dose-dependent manner, potentially contributing to its anti-obesity and metabolic regulatory effects.

### 3.5. Central Administration of KRGM-Gintonin Attenuates HFD-Induced Obesity and Enhances Muscle Function

**Figure 5:**
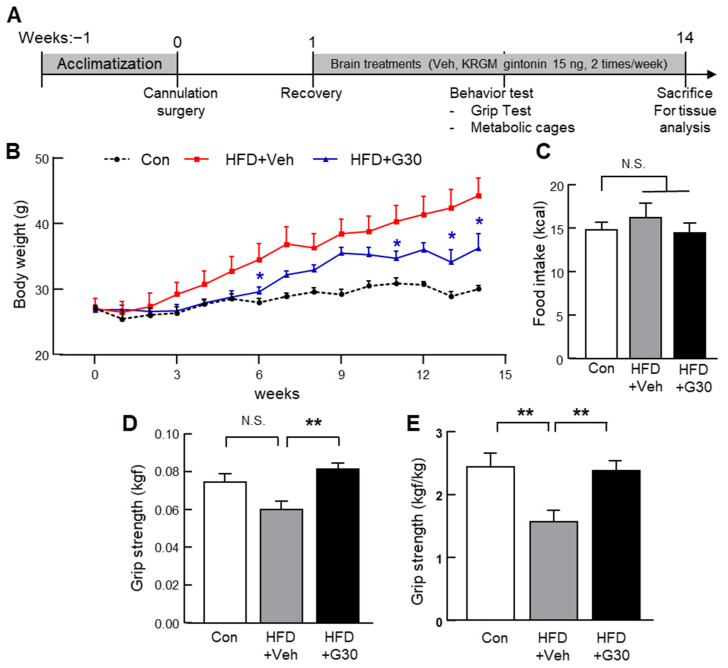
Intracerebroventricular (ICV) injection of KRGM-gintonin reduces body weight and improves muscle strength in HFD-fed mice. (**A**) Schematic timeline of the 14-week experimental protocol. (**B**) Body weight measured weekly throughout the study. (**C**) Daily food intake recorded over a 24 h period. (**D**,**E**) Effect of KRGM-gintonin on normalized forelimb grip strength and grip strength adjusted for body weight, assessed at week 12. Data are expressed as mean ± S.E.M. (*n* = 6–7). Statistical differences between Con, HFD+Veh (PBS injection), and HFD+G30 (KRGM-gintonin, 30 ng/0.5 μL)) were determined using one-way ANOVA followed by Tukey’s post hoc test: ** *p* < 0.01, N.S., not significant. Asterisks in different colors denote statistically significant differences between the corresponding treatment groups, with each color matching the group it represents.

Given the positive metabolic outcomes observed with oral administration of KRGM-gintonin, we next investigated whether central (ICV) administration would exert similar effects. To evaluate the efficacy of centrally delivered KRGM-gintonin in modulating energy balance, C57BL/6N mice (*n =* 6–7) were fed an HFD and received ICV injections of KRGM-gintonin at a dose of 30 ng/0.5 µL, three times per week for 14 consecutive weeks. Body weight and food intake were monitored throughout the experiment. Mice in the HFD group that received PBS exhibited a significant increase in body weight, whereas those treated with ICV KRGM-gintonin showed a marked attenuation of HFD-induced weight gain (Figure 5A,B). These results indicate that central KRGM-gintonin administration effectively suppresses body weight gain associated with HFD feeding. To determine whether the reduction in body weight was attributable to changes in feeding behavior, food intake was measured over a 24 h period. No significant difference in food consumption was observed between groups (Figure 5C), suggesting that the weight-suppressive effect of KRGM-gintonin was not due to altered appetite or caloric intake.

Furthermore, we assessed the effect of ICV KRGM-gintonin on muscle performance using a grip strength test. HFD-fed mice receiving saline injections showed a significant decline in grip strength compared to chow-fed controls. In contrast, KRGM-gintonin-treated HFD mice exhibited a significant improvement in grip strength (Figure 5D,E). Taken together, these findings demonstrate that ICV administration of KRGM-gintonin reproduces the beneficial effects observed with oral administration, reducing body weight and enhancing muscle function, independent of food intake modulation.

### 3.6. ICV Administration of KRGM-Gintonin Enhances Energy Expenditure

**Figure 6:**
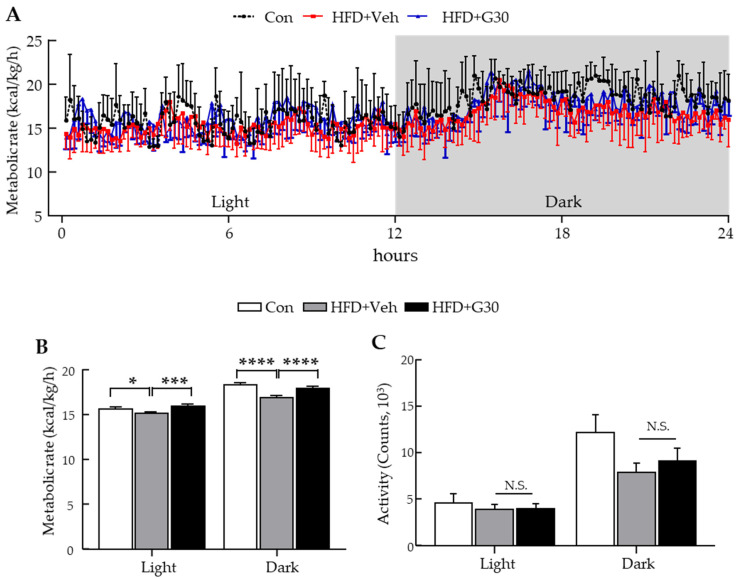
ICV administration of KRGM-gintonin improves energy expenditure and metabolic profile in HFD-fed mice. (**A**) 24 h metabolic rate (kcal/kg/h) calculated from oxygen consumption and carbon dioxide production. (**B**) Average metabolic rate during the experimental period. (**C**) Locomotor activity counts recorded throughout the study. Data are expressed as mean ± S.E.M. (*n* = 6–7). Statistical differences between Con, HFD+Veh and HFD+G30 were determined using one-way ANOVA followed by Tukey’s post hoc test: * *p* < 0.05, *** *p* < 0.001, **** *p* < 0.0001, N.S., not significant.

Given that ICV injection of KRGM-gintonin reduces body weight without affecting food intake, we hypothesized that this effect may be associated with enhanced energy expenditure, especially considering gintonin’s known role in regulating brain function [23]. To test this, we performed indirect calorimetry on mice fed either a CD or HFD, with or without ICV administration of KRGM-gintonin, during both light and dark cycles. The results revealed that ICV KRGM-gintonin significantly elevated metabolic rate across both light and dark periods (Figure 6A,B), similar to the effects observed with oral administration. However, no significant differences in physical activity were detected during the observation period (Figure 6C). Collectively, these findings suggest that both oral and ICV administration of KRGM-gintonin promote energy expenditure and metabolic improvement, thereby contributing to its anti-obesity effects.

## 4. Discussion

Currently, there has been significant focus on researching natural plant compounds that could potentially aid in weight loss and enhance energy expenditure as a means of addressing obesity, as obesity is one of the major global health problems [24]. Ginseng is one such candidate for anti-obesity treatment, as ginseng extracts have been shown to exhibit anti-obesity effects [25,26]. However, the anti-obesity effects of whole ginseng extract remain poorly understood, since it is unclear which ginseng component(s) are actively involved in these effects and how they exert anti-obesity activity at the molecular level [27].

In the present study, we assessed the impact of KRGM-gintonin consumption on the obesity phenotype of HFD-fed mice, focusing on both oral and ICV routes. We demonstrated that long-term KRGM-gintonin treatment alleviates HFD-induced obesity and metabolic syndrome. KRGM-gintonin suppressed body weight gain, increased lean mass, and decreased fat mass without altering food intake [28]. Similarly, ICV administration of KRGM-gintonin exhibited comparable effects on metabolic rate and body weight loss to those observed with oral administration (Figure 5B and Figure 6B).

Oral administration of KRGM-gintonin upregulated thermogenic (UCP1) and lipolytic (HSL) and downregulated anti-inflammatory (TNFα) gene expression in BAT, suggesting activation of pathways associated with BAT function. While previous studies indicate that gintonin can act via LPA-LPA receptors [13,29] this mechanism was not experimentally verified in the current study. Enrichment of lipid components, especially LPA, a potent ligand for G protein-coupled LPA receptors (LPA_1_–_6_), regulates diverse physiological processes, including adipogenesis, thermogenesis, and energy metabolism [13,29]. LPA signaling has been reported to activate BAT and promote the browning of WAT, both of which are associated with increased energy expenditure and reduced fat accumulation [20,30]. LPC, a metabolic precursor of LPA via autotaxin, also exhibits signaling activity and is involved in immune modulation, mitochondrial function, and glucose homeostasis [31]. Our study shows that long-term oral administration of KRGM-gintonin effectively reduces body fat mass gain caused by an HFD by activating thermogenic signaling and increasing metabolic rate in BAT (Figure 4D). BAT is a specialized form of adipose tissue involved in energy metabolism, with mitochondria serving as the primary organelles responsible for this function, as their oxidative capacity directly influences overall metabolic activity. BAT regulates energy usage by stimulating the production of UCP1 in the inner mitochondrial membrane. Enhancing BAT activity and upregulating mitochondrial oxidative phosphorylation proteins can improve mitochondrial function, increase energy expenditure, and ultimately help control obesity and metabolic syndrome [27,32]. UCP1 reduces the proton gradient by uncoupling the respiratory chain and enhances cyclic AMP (cAMP) production, which boosts thermogenesis in mitochondria. Thus, activating UCP1 is a crucial defense mechanism against obesity and related metabolic disorders [6,33,34,35]. Oral administration of KRGM-gintonin upregulated UCP1 mRNA expression (Figure 4D).

Meanwhile, lipolysis is a metabolic process that breaks down stored fats to release energy in response to the body’s energy needs [36]. Lipolysis involves the hydrolysis of triglycerides into fatty acids and glycerol, mediated by lipases such as adipose triglyceride lipase (ATGL) and HSL. HSL is the primary enzyme responsible for breaking down triglycerides deposited in adipose tissue [20,37]. PC also supports lipid transport and hepatic lipoprotein assembly and has been reported to ameliorate diet-induced hepatic steatosis [38]. Oral treatment with KRGM-gintonin significantly upregulated HSL lipolytic gene expression in BAT but not WAT (Figure 4D). In addition, oral treatment with KRGM-gintonin significantly decreased TNF-α gene expression in BAT but not WAT (Figure 4E), indicating that oral administration of KRGM-gintonin may exert dual actions in BAT, including simultaneous activation of lipolytic, thermogenic, and anti-inflammatory pathways (Figure 4D). In this study, we experimentally analyzed UCP1 as a key thermogenic marker, HSL as a lipolytic marker, and TNFα as an inflammatory/cytokine regulator in brown adipose tissue (BAT). While other thermogenic markers (DIO2, PRDM16, CITED1, TBX1), lipolytic markers (ATGL), and additional inflammatory/cytokine regulators (GAB2, IL1β, IL6) have been reported in the literature [39], we did not measure them experimentally; their relevance is discussed to provide broader context for BAT thermogenesis and lipolysis. PPARγ functions primarily as an adipogenesis-related transcription factor [40]. Incorporating these literature-based markers strengthens interpretation while keeping the experimental focus on UCP1, HSL, and TNFα.

KRGM-gintonin-mediated metabolic activation via lipolytic and thermogenic pathways may be associated with improvements in fatty liver and behavioral activities. Oral administration of KRGM-gintonin improves fatty liver by inhibiting hepatic fat accumulation, which is also linked to attenuation of metabolic syndrome markers such as TG and TC (Figure 4A,B). Moreover, although oral KRGM-gintonin had no effect on food intake, supplementation alongside an HFD led to marked improvements in physical activity parameters, including treadmill endurance, running speed, and grip strength, compared with HFD-fed controls (Figure 2A,B,E,F). The observed improvements in treadmill duration, running speed, and grip strength may be partially attributable to BAT-mediated systemic metabolic effects. Enhanced BAT thermogenesis increases energy expenditure and releases batokines such as FGF21, NRG4, and IL-6, which can promote skeletal muscle oxidative metabolism and endurance performance, even without direct changes in WAT or muscle tissue [41]. The higher concentration of these lipids in KRGM-gintonin compared with root- or stem-derived gintonin suggests that the marc-based fraction possesses a more potent pharmacological profile.

In addition to oral treatment, ICV delivery was employed to investigate the direct central effects of KRGM-gintonin on hypothalamic regulation of energy homeostasis. Gintonin is a lysophosphatidic acid (LPA) receptor ligand, and multiple LPA receptor subtypes (LPA_1_–_6_) are highly expressed in brain regions such as the hypothalamus and hippocampus, which are critically involved in appetite control, thermogenesis, and sympathetic outflow to brown adipose tissue (BAT) [22]. Because systemic administration (e.g., intraperitoneal injection) may have limited brain penetration due to the blood–brain barrier, ICV injection allows direct assessment of central gintonin actions. Consistent with previous reports describing central metabolic effects of LPA signaling, ICV administration of KRGM-gintonin reduced body weight and increased metabolic rate (Figure 5D,E and Figure 6B), suggesting potential activation of hypothalamic pathways regulating energy expenditure and BAT thermogenesis. Although neural endpoints were not evaluated in this study, future work will be necessary to delineate the specific hypothalamic circuits and receptor mechanisms mediating these central effects. Taken together, the observed anti-obesity effects of KRGM-gintonin via both oral and central administration routes may, at least in part, be attributed to its enrichment of lipid components, especially LPA, a potent ligand for G protein-coupled LPA receptors (LPA_1_–_6_), which regulates diverse physiological processes, including adipogenesis, thermogenesis, and energy metabolism.

Although our findings provide novel insight into the anti-obesity potential of KRGM-gintonin, several limitations should be acknowledged. First, we did not include molecular mechanism data (e.g., Western blotting for UCP1, p-AMPK, or LPA receptor signaling) to confirm the mechanistic pathways of KRGM-gintonin via oral or ICV routes. However, previous studies have demonstrated a strong correlation between UCP1 mRNA levels and protein expression and thermogenic function [42], supporting our interpretation that the observed increase in UCP1 mRNA reflects enhanced BAT thermogenic activity. Although cold tolerance was not tested, upregulated UCP1 mRNA generally correlates with enhanced thermogenic capacity and mitochondrial activity. Future studies using hepatocyte models and AMPK knockdown (e.g., siRNA) approaches will be critical to confirm whether KRGM-gintonin directly activates AMPK-dependent signaling pathways. Furthermore, intraperitoneal glucose tolerance test (IPGTT) data were not included, as oral administration of KRGM-gintonin did not significantly improve glucose clearance compared with HFD (Figure S2). In contrast, ICV administration of KRGM-gintonin markedly enhanced glucose tolerance relative to HFD-fed mice, suggesting the involvement of a central regulatory mechanism. These preliminary findings highlight a potential central mode of action and warrant further investigation into the CNS-mediated metabolic effects of KRGM-gintonin. Additionally, KRGM-gintonin is a newly characterized extract, and long-term treatment was administered intermittently without optimizing dosing frequency or duration. Future studies should explore the molecular mechanisms underlying its central vs. peripheral effects using Western blotting and immunohistochemistry. Furthermore, comprehensive metabolic profiling, gut microbiota analysis, and behavioral assessments could provide deeper insight into its systemic impact to fully establish its therapeutic potential in obesity research.

## 5. Conclusions

In summary, oral and ICV administration of KRGM-gintonin modulated gene expression associated with thermogenesis and lipid metabolism in HFD-induced obese mice, suggesting activation of BAT-related pathways. These effects are likely mediated by LPA-enriched bioactive lipids acting on central and peripheral LPA receptors. Future studies incorporating protein-level validation, histological analysis, and functional metabolic assays are warranted to confirm these mechanisms.

## Figures and Tables

**Table 1:** Comparison of lipid component content (% *w*/*w*) between gintonin-enriched fraction (GEF) and Korean red ginseng marc (KRGM)-gintonin.

Lipid Component	Gintonin-Enriched Fraction (% *w*/*w*)	KRGM-Gintonin (% *w*/*w*)
Lysophosphatidic acid C18:2	~0.20%	~0.27%
Lysophosphatidylcholine C18:2	~0.082%	~0.99%
Phosphatidylcholine C16:0–18:2	~0.028%	~1.38%
Phosphatidylcholine C18:2–18:2	~0.23%	~1.48%

**Table 2:** Primer sequences.

Name	Forward	Reverse
UCP1	TCTCAGCCGGCTTAATGACT	TGCATTCTGACCTTCACGAC
PGC1-α	GTCAACAGCAAAAGCCACAA	GTGTGAGGAGGGTCATCGTT
PPAR-γ	ACGATCTGCCTGAGGTCTGT	CATCGAGGACATCCAAGACA
PPAR-α	TCGGACTCGGTCTTCTTGAT	TCTTCCCAAAGCTCCTTCAA
PPAR-β	TGGAGCTCGATGACAGTGAC	GGTTGACCTGCAGATGGAAT
TNF-α	CAGGCGGTGCCTATGTCTC	CGATCACCCCGAAGTTCAGTAG
Gab2	TCTGAGACTGATAACGAGGAT	GATGGAGTCGGCTGTTG
IL-1β	AGATGAAGGGCTGCTTCCAAA	GGAAGGTCCACGGGAAAGAC
IL-6	CTGCAAGAGACTTCCATCCAG	AGTGGTATAGACAGGTCTGTTGG
ATGL	CAACGCCACTCACATCTACGG	GGACACCTCAATAATGTTGGCAC
HSL	CCCCTGCGACGATTATCAAGA	CAGTGGCTGATGCAGTTATGTT
AMPK	GTGGTGTTATCCTGTATGCCCTTCT	CTGTTTAAACCATTCATGCTCTCGT
β-actin	CTCCGGCATGTGCAA	CCCACCATCACACCCT

## Data Availability

The original contributions presented in this study are included in the article and Supplementary Materials. Further inquiries can be directed to the corresponding author.

## Supplementary Materials

The following supporting information can be downloaded at: https://www.mdpi.com/article/10.3390/nu17233794/s1, Table S1: Composition of the 60%kcal high-fat diet; Table S2: Oral administration mouse body weight; Table S3: Analysis results of mouse body composition; Table S4: KRGM-gintonin treatment improved plasma lipid profile; Table S5: Relative mRNA expression of brown adipose tissue; Table S6: The mRNA expression levels in white adipose tissue (WAT) showed no statistically significant difference between the groups; Table S7: KRGM-gintonin treatment significantly increased the metabolic rate; Figure S1: Effect of oral KRGM-gintonin administration on food intake in HFD-fed mice; Figure S2: Effects of KRGM-gintonin on glucose tolerance in HFD-fed mice.

## Abbreviations

ATGL	adipose triglyceride lipase
BAT	brown adipose tissue
CD	chow diet
FFA	free fatty acid
GEF	gintonin-enriched fraction
HFD	high-fat diet
HSL	hormone-sensitive lipase
ICV	intracerebroventricular
KRGM-gintonin	Korean red ginseng marc-derived gintonin
LPA	lysophosphatidic acid
LPC	lysophosphatidylcholine
PGC1α	Peroxisome proliferator-activated receptor gamma coactivator 1-α
PRDM16	PRD1-BF1-RIZ1 homologous domain containing 16
TA	tibialis anterior
TC	total cholesterol
TG	triacylglycerol
TNF-α	tumor necrosis factor-alpha
UCP1	uncoupling protein-1
WAT	white adipose tissue
