# Supplementary material for: Lipid-Enriched Gintonin from Korean Red Ginseng Marc Alleviates Obesity via Oral and Central Administration in Diet-Induced Obese Mice
Source: Nutrients. 2025 Dec 3;17(23):3794. doi: 10.3390/nu17233794 (PMC12694193; doi:10.3390/nu17233794)

Supplementary table S1. Composition of the 60%kcal high-fat diet.

Class description	Ingredients	grams	percent
Protein	Casein, Lactic, 30 Mesh	200 g	25.8%
Protein	Cystine, L	3 g	0.4%
Carbohydrate	Lodex 10	125 g	16.2%
Carbohydrate	Sucrose, Fine Granulated	72.8 g	9.4%
Fiber	Solka Floc, FCC200	50 g	6.5%
Fat	Lard	245 g	31.7%
Fat	Soybean Oil, USP	25 g	3.2%
Mineral	S10026B	50 g	6.5%
Vitamin	Choline Bitartrate	2 g	0.3%
Vitamin	V10001C	1 g	0.1%
Dye	Dye, Blue FD&C #1, Alum. Lake 35-42%	0.05 g	0.0%
	Total:	773.85 g	100%

Supplementary table S2. Oral administration mouse body weight. Values that do not share a common superscript letter (a, b, c) are statistically significantly different (Tukey's post hoc test, $p<0.05$)

		Body weight	
	n	0 week	25 weeks
Con	10	20.25 ±0.45	27.72 ±0.47 [c]
HFD+Veh	11	20.32 ±0.28	47.94 ±1.21 [a]
HFD+G50	10	20.31 ±0.26	46.69 ±0.79 [ab]
HFD+G200	10	20.31 ±0.25	44.44 ±0.85 [b]

Supplementary table S3. Analysis results of mouse body composition. Different superscript letters indicate statistically distinct groups between mouse groups following post-hoc analysis (Tukey's post hoc test, *p*<0.05).

	**Lean mass (%)**	**Fat mass (%)**
Con	91.14±0.97^{a}	6.945±0.87^{b}
HFD+Veh	53.44±1.14^{c}	45.81±1.19^{a}
HFD+G50	56.63±1.17^{bc}	42.05±1.14^{a}
HFD+G200	57.75±0.64^{b}	43.42±1.14^{a}

Supplementary table S4. KRGM-gintonin treatment improved plasma lipid profile. Different superscript letters indicate a statistically significant difference between groups (Tukey's post hoc test, $p<0.05$).

	**CD**	**HFD**	**HFD+G50**	**HFD+G200**
TG	47.62 ±2.732^{b}	83.76 ±10.54^{a}	56.39 ±2.782^{b}	57.29 ±4.604^{b}
TC	67.86 ±2.962^{c}	226 ±11.10^{a}	198.2 ±5.389^{ab}	182.1 ±4.726^{b}
FFA	0.7856 ±0.0318^{a}	0.9926 ±0.0858^{a}	0.9611 ±0.0411^{a}	0.9498 ±0.0691^{a}

Supplementary table S5. Relative mRNA expression of brown adipose tissue. Data points are only considered statistically comparable when they share a common superscript letter (a, b).

BAT	CD	HFD	HFD+G50	HFD+G200
UCP1	1.000 ±0.2142^{ab}	0.4248 ±0.0312^{a}	0.7735 ±0.1533^{ab}	1.341 ±0.2128^{b}
PGC-1α	1.000 ±0.2459	0.1878 ±0.0296	0.3705 ±0.0876	0.2823 ±0.0618
PPARγ	1.000 ±0.1625	0.4777 ±0.0793	0.5928 ±0.1279	0.608 ±0.1026
ATGL	1.000 ±0.2205	0.6962 ±0.0933	0.4822 ±0.1094	0.817 ±0.1975
HSL	1.000 ±0.06^{ab}	0.685 ±0.036^{a}	1.055 ±0.1408^{b}	0.9495 ±0.0493^{ab}
TNF-α	1.000 ±0.2317^{ab}	1.625 ±0.1103^{b}	1.365 ±0.0647^{ab}	0.7876 ±0.1079^{a}
GAB-2	1.000 ±0.2267	0.7818 ±0.0622	0.9642 ±0.265	1.011 ±0.193
IL-1β	1.000 ±0.2238	0.3937 ±0.0773	0.3583 ±0.0711	0.2153 ±0.0255
IL-6	1.000 ±0.4111	0.4742 ±0.0617	0.6266 ±0.1467	0.3648 ±0.0661

Supplementary table S6. The mRNA expression levels in white adipose tissue (WAT) showed no statistically significant difference between the groups.

WAT	CD	HFD	HFD+G50	HFD+G200
UCP1	1.000 ±0.4778	0.0194 ±0.0058	0.0619 ±0.0245	0.0321 ±0.024
PGC1α	1.000 ±0.3725	0.104 ±0.0187	0.1465 ±0.0476	0.0603 ±0.0397
PPARγ	1.000 ±0.1811	0.4019 ±0.0371	0.3907 ±0.0634	0.3789 ±0.0717
PPAR-α	1.000 ±0.4452	0.1846 ±0.0243	0.1592 ±0.0357	0.2563 ±0.2563
PPAR-β	1.000 ±0.351	0.374 ±0.0188	0.4397 ±0.0615	0.3699 ±0.0537
PPAR-γ	1.000 ±0.2427	0.2203 ±0.0158	0.2076 ±0.0435	0.2343 ±0.0384
AMPKα	1.000 ±0.2455	0.1373 ±0.1373	0.166 ±0.0435	0.1728 ±0.0462
ATGL	1.000 ±0.345	0.685 ±0.0296	0.4566 ±0.0932	0.5224 ±0.0989
HSL	1.000 ±0.2111	0.6664 ±0.0856	0.5993 ±0.0896	0.5627 ±0.2047
GAB-2	1.000 ±0.3299	1.312 ±0.1553	1.206 ±0.2698	0.8501 ±0.1928
TNF-α	1.000 ±0.2902	0.4856 ±0.066	0.7358 ±0.1892	0.7067 ±0.0872
IL-1β	1.000 ±0.3299	1.168 ±0.1432	0.8363 ±0.1762	0.7077 ±0.1537
IL-6	1.000 ±0.2902	0.5263 ±0.0675	0.6698 ±0.168	0.7067 ±0.0872

Supplementary table S7. KRGM-gintonin treatment significantly increased the metabolic rate. Different superscript letters indicate statistically distinct groups between mouse groups following Tukey's post hoc test analysis (p<0.05).

	**Con**	**HFD+Veh**	**HFD+G50**	**HFD+G200**
Light	14.156 ±0.3494[a]	11.5335 ±0.2856[d]	12.2156 ±0.6916[c]	13.5533 ±0.3605[b]
Dark	17.2229 ±0.4201[a]	12.9240 ±0.2687[d]	13.7371 ±0.7183[c]	15.5173 ±0.3401[b]

Supplementary figure S1. Effect of oral KRGM-gintonin administration on food intake in HFD-fed mice. Food intake was measured over 24 h on four separate days across the 3-month treatment period. No significant differences were observed in cumulative or average food intake among the experimental groups. Data are presented as mean ± SEM.

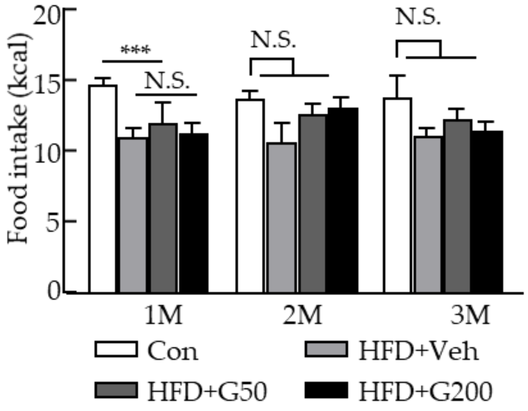

Supplementary figure S2. Effects of KRGM-gintonin on glucose tolerance in HFD-fed mice. (A) Oral administration of KRGM-gintonin did not significantly affect systemic glucose tolerance. (B) Central administration via ICV injection significantly improved glucose homeostasis. Data are presented as mean ± SEM; statistical significance is indicated where applicable.

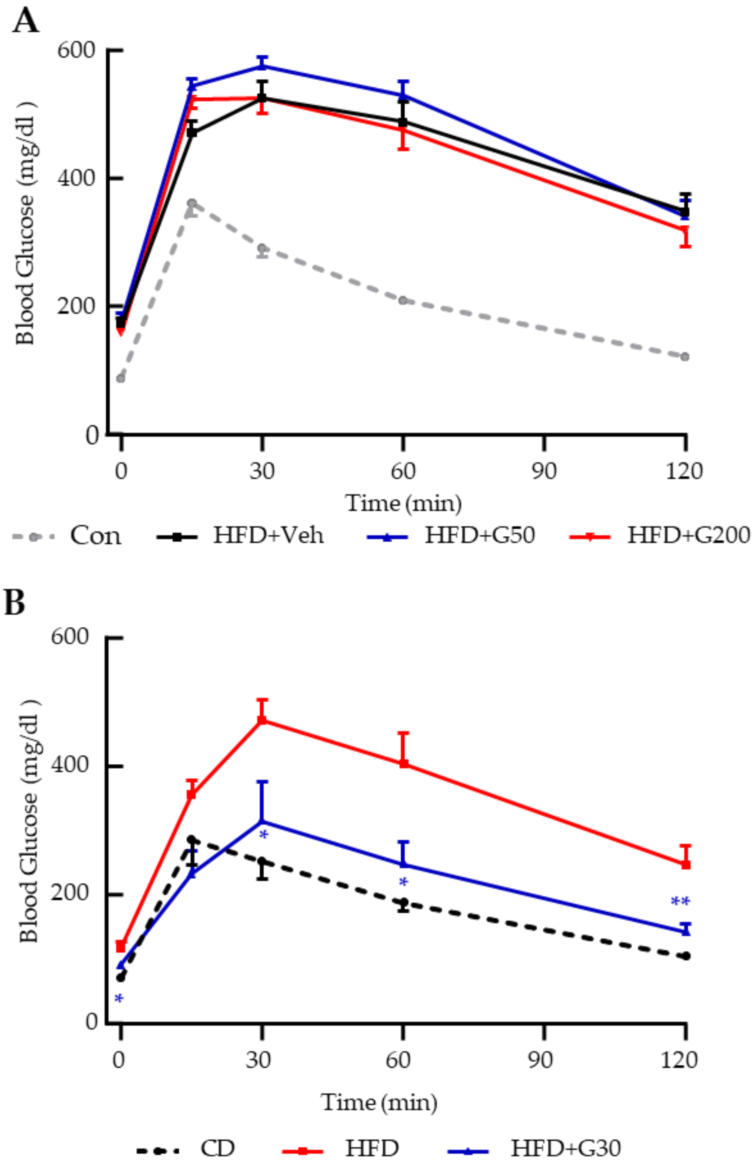

Supplement: Supplementary file 1 [file nutrients-17-03794-s001.zip › nutrients-3918644-supplementary.pdf]
